# Proteome signatures reveal homeostatic and adaptive oxidative responses by a putative co-chaperone, Wos2, to influence fungal virulence determinants in cryptococcosis

**DOI:** 10.1128/spectrum.00152-24

**Published:** 2024-07-02

**Authors:** Brianna Ball, Arjun Sukumaran, Samanta Pladwig, Samiha Kazi, Norris Chan, Effie Honeywell, Manuela Modrakova, Jennifer Geddes-McAlister

**Affiliations:** 1 Department of Molecular and Cellular Biology, University of Guelph, Guelph, Ontario, Canada; University of Michigan Michigan Medicine, Ann Arbor, Michigan, USA

**Keywords:** quantitative proteomics, fungal pathogenesis, *Cryptococcus neoformans*, infection, virulence, co-chaperone, *in vivo *models

## Abstract

The increasing prevalence of invasive fungal pathogens is dramatically changing the clinical landscape of infectious diseases, posing an imminent threat to public health. Specifically, *Cryptococcus neoformans*, the human opportunistic pathogen, expresses elaborate virulence mechanisms and is equipped with sophisticated adaptation strategies to survive in harsh host environments. This study extensively characterizes Wos2, an Hsp90 co-chaperone homolog, featuring bilateral functioning for both cryptococcal adaptation and the resulting virulence response. In this study, we evaluated the proteome and secretome signatures associated with *wos2* deletion in enriched and infection-mimicking conditions to reveal Wos2-dependent regulation of the oxidative stress response through global translational reprogramming. The *wos2*Δ strain demonstrates defective intracellular and extracellular antioxidant protection systems, measurable through a decreased abundance of critical antioxidant enzymes and reduced growth in the presence of peroxide stress. Additional Wos2-associated stress phenotypes were observed upon fungal challenge with heat shock, osmotic stress, and cell membrane stressors. We demonstrate the importance of Wos2 for intracellular lifestyle of *C. neoformans* during *in vitro* macrophage infection and provide evidence for reduced phagosomal replication levels associated with *wos2*Δ. Accordingly, *wos2*Δ featured significantly reduced virulence within impacting fungal burden in a murine model of cryptococcosis. Our study highlights a vulnerable point in the fungal chaperone network that offers a therapeutic opportunity to interfere with both fungal virulence and fitness.

**IMPORTANCE:**

The global impact of fungal pathogens, both emerging and emerged, is undeniable, and the alarming increase in antifungal resistance rates hampers our ability to protect the global population from deadly infections. For cryptococcal infections, a limited arsenal of antifungals and increasing rates of resistance demand alternative therapeutic strategies, including an anti-virulence approach, which disarms the pathogen of critical virulence factors, empowering the host to remove the pathogens and clear the infection. To this end, we apply state-of-the-art mass spectrometry-based proteomics to evaluate the impact of a recently defined novel co-chaperone, Wos2, toward cryptococcal virulence using *in vitro* and *in vivo* models of infection. We explore global proteome and secretome remodeling driven by the protein and uncover the novel role in modulating the fungal oxidative stress response. Complementation of proteome findings with *in vitro* infectivity assays demonstrated the protective role of Wos2 within the macrophage phagosome, influencing fungal replication and survival. These results underscore differential cryptococcal survivability and weakened patterns of dissemination in the absence of *wos2*. Overall, our study establishes Wos2 as an important contributor to fungal pathogenesis and warrants further research into critical proteins within global stress response networks as potential druggable targets to reduce fungal virulence and clear infection.

## INTRODUCTION

*Cryptococcus neoformans* is an opportunistic fungal pathogen responsible for cryptococcosis, a highly invasive and difficult-to-manage disease. This disease currently affects over 230,000 individuals annually and results in 19% of AIDS-related mortality (1, 2). Invasive human fungal pathogens are equipped with important virulence determinants for survival within the adverse mammalian host environment, which challenge pathogens with an elevated body temperature, effective immune system, and nutrient deprivation. Recently, we applied mass spectrometry (MS)-based proteomics to investigate the adaptation of *C. neoformans* under nutrient limitation of the essential metal, zinc (3). We observed zinc-associated regulation of Wos2, an uncharacterized Hsp90 co-chaperone homolog; our *in vitro* characterization of the protein defined a novel role in virulence factor modulation (i.e., thermotolerance, zinc utilization, melanin pigmentation, and capsule production).

The role of co-chaperones in fungal virulence is well-supported by heat shock protein (HSP) characterization as primary responders to environmental stress (4–7). The relationship between HSPs and fungal virulence is well-represented by Hsp70s and Hsp90s (8). For example, *C. neoformans* depend on Hsp90 machinery for thermotolerance and fluconazole susceptibility, and Hsp70 is involved in melanin pigmentation (5, 9, 10). Additionally, proteomic analysis revealed that packaging of both Hsp70 and Hsp90 into extracellular vesicles along with a myriad of other pathogenesis-related molecules resulted in the construction of “virulence bags” capable of delivering pathogenic cargo across the body during infection (4, 6, 11). In comparison, Hsp90 of *Candida albicans* is vital in commanding yeast-to-hyphal transitions and regulating drug resistance (12–14). However, HSPs do not function independently; they rely on a complex network of proteins to facilitate client protein folding and modifications. For instance, co-chaperones in the Hsp90 system feature polarizing control over Hsp90-mediated client protein folding (15).

Our study investigates *C. neoformans* Wos2, which presents substantial homology to the conserved p23 co-chaperone expressed widely across eukaryotes (16). p23 features considerable chaperone-independent functions, including ribosome biogenesis, Golgi operations, and DNA repair activities (5), and it is a central component of the Hsp90 machinery (8, 17, 18). Hsp90 interacts with approximately 10% of all yeast proteins; thus, impairment of the Hsp90 network features an opportunity for detrimental functioning to multiple branches of the fungal stress and virulence response network (5, 18, 19). For instance, defined interactions between Hsp90 and a co-chaperone, Sgt1, orchestrate morphogenesis and drug resistance in the human fungal pathogen, *C. albicans* (20). In addition, recent studies highlighted the critical role of p23 in stabilizing the complex formed by Hsp90 with client proteins, demonstrating that p23 is essential for general Hsp90 functioning, regardless of the respective client protein (15, 21). Moreover, in the model fungal organism, *Neurospora cassa,* deletion of the Hsp90 co-chaperones, p23, Sti1, and Aha1, resulted in hypersensitivity to azoles and heat (22).

Given the importance of co-chaperones across a spectrum of fungal pathogens and their association to antifungal susceptibility, the characterization of novel co-chaperones is an important avenue of discovery to regulate the prevalence and outcome of fungal infections. This study explores the connections between co-chaperone, virulence, and anti-virulence targets using high-resolution quantitative proteomics profiling. We reveal extensive proteome and secretome remodeling during infection-mimicking conditions compared to enriched environments, indicating the requirement for Wos2 to drive intra- and extracellular homeostasis during infection-related stress. We established that Wos2 regulates the response of *C. neoformans* to oxidative stress and adaptation during prolonged infectious states within macrophages, influencing fungal replication and survival. Furthermore, this is the first report of *wos2* establishing fungal virulence, which, upon deletion, significantly attenuates a murine model of cryptococcosis, further supporting the protein as a novel target with therapeutic intervention potential.

## RESULTS

### Culture conditions promote cellular proteome remodeling for *wos2*Δ

**Fig 1:**
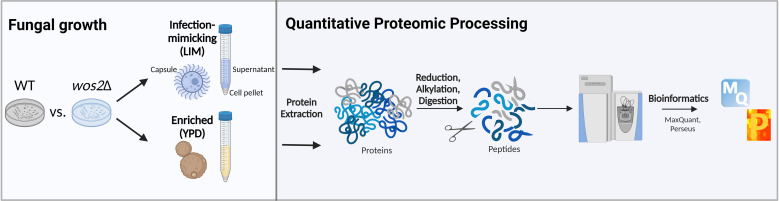
Workflow for mass spectrometry-based proteomics profiling. *C. neoformans* WT and *wos2*∆ strains were cultured in infection-mimicking (low-iron media, LIM) or enriched (yeast peptone dextrose, YPD) media and subjected to our total and secretome protein extraction protocol (e.g., sonication with detergent), followed by protein reduction, alkylation, and digestion and measurement by mass spectrometry. Data analysis, visualization, and statistical processing were performed using MaxQuant and Perseus (25, 26). The experiment was completed in biological quadruplicate. Figure was generated with Biorender.com.

To gain insights into the global alterations that occur upon deletion of *wos2* on the *C. neoformans* proteome, we compared protein production profiles between *C. neoformans* wild-type (WT) and a *wos2*Δ strain. We investigated the connection between Wos2 and fungal adaptation to a host-like environment by profiling the protein-level changes within the cellular proteome (cell pellet) of cells grown to mid-log phase under enriched ( yeast-peptone-dextrose media, YPD) and infection-mimicking ( low-iron media, LIM) conditions (Fig. 1) (23, 24).

**Fig 2:**
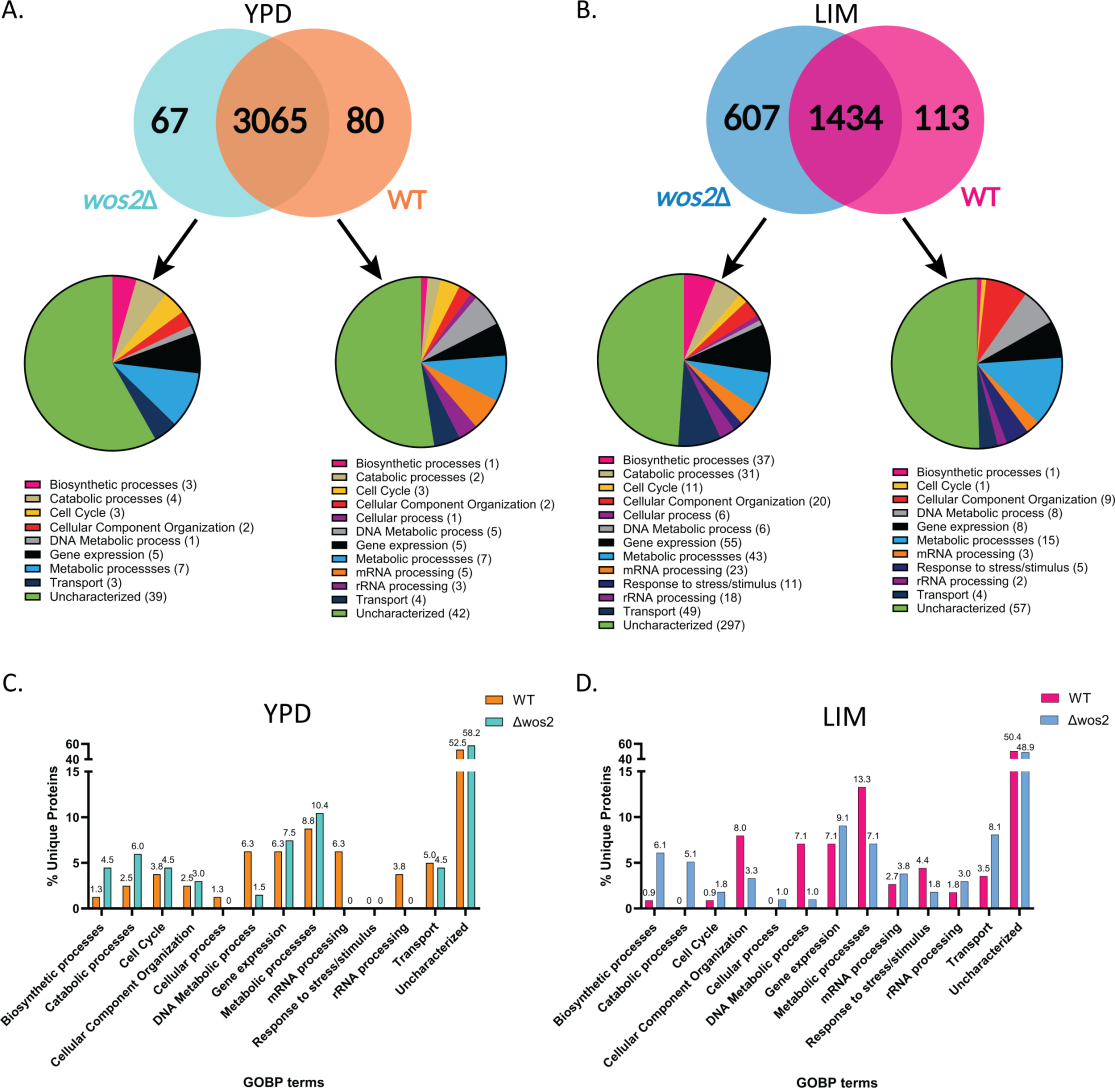
Wos2-dependent cellular proteome signatures between enriched and infection-mimicking environments. (**A**) Venn diagram for the number of unique proteins identified in the YPD (enriched) cellular proteome between *C. neoformans* WT (orange; 80) and *wos2*∆ (turquoise; 67) strains with 3,065 proteins commonly identified. The distribution of Gene Ontology biological processes (GOBP) terms for identified unique fungal proteins are shown exclusive to each strain. (**B**) Venn diagram for the number of unique proteins identified in the LIM (infection-mimicking) cellular proteome between *C. neoformans* WT (pink; 113) and *wos2*∆ (blue; 607) strains with 1,434 proteins commonly identified. (**C**) Comparison of the percentage of unique proteins exclusive to each strain identified in the YPD cellular proteome and distributed according to GOBP terms; percentage of GOBP terms indicated on top of the respective bar. (**D**) Comparison of the percentage of unique proteins exclusive to each strain identified in the LIM cellular proteome and distributed according to GOBP terms; percentage of GOBP terms indicated on top of the respective bar. The experiment was performed in biological quadruplicate. YPD: yeast peptone dextrose; LIM: low-iron media.

Our analysis detected 3,420 unique proteins (2,933 proteins after valid value filtering) across the samples, representing approximately 46% of the *C. neoformans* proteome. The YPD-enriched proteome consisted of 3,065 proteins shared between WT and *wos2*∆, whereas 80 proteins were detected only in the WT proteome and 67 proteins identified solely within *wos2*∆ (Fig. 2A). Conversely, the infection-mimicking proteome consisted of 1434 proteins identified from both strains, whereas the WT and *wos2*∆ proteomes identified 113 and 607 unique proteins, respectively (Fig. 2B). Next, we classified the biological attributes of the unique proteins identified within each group using Gene Ontology of biological processes (GOBP). Enriched growth conditions revealed a balanced abundance of proteins between the WT and *wos2*Δ strains across multiple categories, including cell cycle, gene expression, transport, and uncharacterized roles, with the detection of proteins unique to WT associated with mRNA and rRNA (Fig. 2C). Similarly, infection-mimicking conditions revealed a consistent representation of protein categories between the strains, including an anticipated increase in stress and stimulus response proteins under nutrient-limited conditions (Fig. 2D).

**Fig 3:**
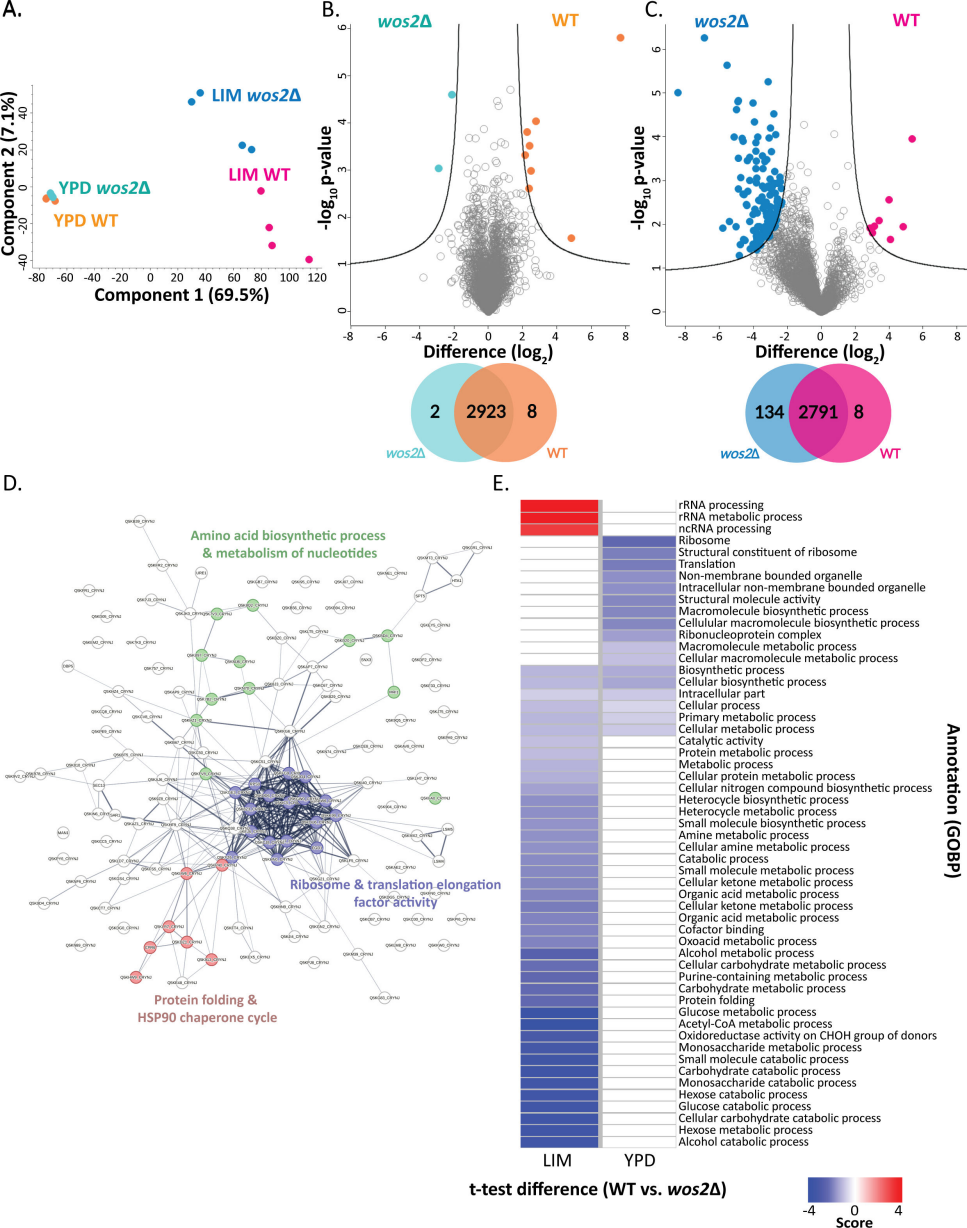
Wos2 remodeling of the global fungal proteome. (**A**) Principal component analysis for YPD (enriched) and LIM (infection-mimicking) conditions. (**B**) Volcano plot comparing proteins identified under enriched (i.e., YPD) fungal growth between WT and *wos2*∆. Highlighted proteins depict significant changes in protein abundance upon strain comparison; Venn diagram depicts the number of significantly different proteins identified in each condition. (**C**) Volcano plot comparing proteins identified under infection-mimicking (i.e., LIM) conditions between WT and *wos2*∆. Highlighted proteins depict significant changes in protein abundance upon strain comparison; Venn diagram depicts the number of significantly different proteins identified in each condition. Student’s *t* test, *P* < 0.05; FDR = 0.05; S_0_ = 1. (**D**) STRING analysis for fungal proteins with a significant increase in abundance within *wos2*∆ under infection-mimicking conditions (LIM). Color coding for network nodes grouped within local STRING network clusters (i.e., protein folding and Hsp90 cycle for steroid hormone receptors in the presence of a ligand, structural constituent of ribosome and translation elongation factor activity, and amino acid biosynthetic process and metabolism of nucleotides) included in the figure, and the thickness of the lines connecting network edges indicates the confidence of the data support. PPI enrichment *P*-value = 7.36e-06; functional clusters enriched with FDR = 0.05. (**E**) 1D annotation enrichment for proteins defined within categories based on Gene Ontology biological processes (GOBP). Student’s *t* test, *P* < 0.05; FDR = 0.05. Experiment performed in biological quadruplicate.

We next assessed drivers of protein-level differences across the strains and growth conditions. A principal component analysis (PCA) of the cellular proteome indicated the largest component of separation as fungal growth conditions (i.e., YPD vs LIM) (component 1, 69.5%), with the second component of separation attributed to the absence of *wos2* (component 2, 7.1%) (Fig. 3A). A comparison of significant differences in protein abundance between WT and *wos2*∆ under enriched conditions defined eight proteins with significantly higher abundance in WT and two proteins with significantly higher abundance in the mutant (Fig. 3B; Table S1). Importantly, Wos2 (CNAG_07558) was >7.5 fold (log_2_) in the WT strain compared to the mutant strain, confirming disruption. We also observed a significant increase in abundance of proteins within the WT strain important for reactive oxygen species (ROS) detoxification, including the well-characterized antioxidant defense protein, catalase 3 [Cat3, CNAG_00575; >2.4 fold [log_2_]), as well as a predicted peroxin protein [CNAG_03394; >4.5 fold [log_2_]), with proposed peroxisomal localization, an organelle essential for sequestering diverse oxidative reactions (27–29). Furthermore, four uncharacterized proteins (CNAG_04585; >2.1 fold [log_2_], CNAG_01387; >2.3 fold [log_2_], CNAG_00848; >2.3 fold [log_2_], and CNAG_03966; >2.7 fold [log_2_]) were more abundant in the WT strain along with nuclease I (CNAG_00264; >2.2 fold [log_2_]) involved in the DNA catabolic process. Conversely, in the *wos2*∆ strain, two uncharacterized membrane-bound proteins were more abundant, including an uncharacterized protein involved in transport to the plasma membrane (CNAG_07020; >2.1 fold [log_2_]) and a protein with predicted phospholipid biosynthesis activity (CNAG_04522; >2.9 fold [log_2_]), suggesting alterations in the cellular organization of both phospholipid membranes and their proteinaceous components in the absence of Wos2.

We previously reported the important role of Wos2 in *C. neoformans* classical virulence factor production, i.e., capsule and melanin production and thermotolerance in zinc limitation (3); therefore, given the similar overlapping YPD-enriched proteomic signatures of the strains, we next assessed if virulence-inducing stress promotes distinct proteome changes in the absence of *wos2*. In this study, we observed a significant increase in the production of eight proteins in the WT strain and a vast difference in the mutant strain, with 134 proteins showing significantly higher production (Fig. 3C; Table S1). In the WT strain, proteins featured a wide range of functions within cellular respiration, including an NADH-ubiquinone oxidoreductase subunit (CNAG_05267; >3 fold [log_2_]) of the electron transport chain (ETC), a mitochondrial pyruvate carrier (CNAG_00092; >3.3 fold [log_2_]), and Oxa1 (CNAG_02769; >4.8 fold [log_2_]), which mediates the assembly of components within the ETC (30). Notably, across both enriched and infection-mimicking conditions, an uncharacterized protein (CNAG_00848; >2.3 fold [log_2_]) was consistently more abundant within the WT strain, with predicted nuclear chaperone and ribosomal biogenesis functions (31, 32).

The protein with the greatest difference in abundance within *wos2*Δ was the well-defined cytokine-inducing glycoprotein Cig1 (>8.4 fold [log_2_]), an established *C. neoformans* virulence-associated hemophore crucial for iron acquisition and production in iron-starved cells (i.e., under LIM growth conditions), serving as a robust positive control for our study (33). We also report an increased abundance in the *wos2*Δ strain of eIF2α (CNAG_07778; >3.8 fold [log_2_]) and eIF2β (CNAG_04269; >3.2 fold [log_2_]); both proteins function in coordination for initiation of translation and stress adaptation in *C. neoformans* (34–36). Next, we defined an interconnected protein–protein association network with designated protein clusters (i.e., STRING local network clusters) for the significantly altered *wos2*Δ proteome. We observed enrichment in protein folding and Hsp90 cycle for steroid hormone receptors in the presence of a ligand, structural constituent of ribosome and translation elongation factor activity, and amino acid biosynthetic process and metabolism of nucleotides (37) (Fig. 3D).

Given the diverse impact of *wos2* deletion on the proteome under altered media conditions, we performed a 1D annotation enrichment (38) to comprehensively characterize the influence of Wos2 based on GOBP. Under YPD conditions, we observed enrichment of proteins within *wos2*Δ associated with the ribosome, translation, structural activity, and biosynthetic and metabolic processes (Fig. 3E); no enrichment of categories for the WT was observed. Under infection-mimicking (LIM) conditions, we observed broader enrichment across protein categories, including rRNA processing, metabolic process, and ncRNA processing for WT. Conversely, for *wos2*Δ, we observed enrichment across 41 categories with an emphasis on molecular metabolic (i.e., hexose, monosaccharide, acetyl-CoA, and glucose) and catabolic (i.e., alcohol, carbohydrate, and glucose) processes, protein metabolic process and folding, and oxidoreductase activity. Together, these data support Wos2 as a central player in fungal adaptation during stress- and infection-mimicking conditions indicated by remodeling at the protein level to drive such adaptations.

### Wos2 supports fungal adaptation to osmotic and cell membrane stress and drives tolerance to elevated oxidative stress

**Fig 4:**
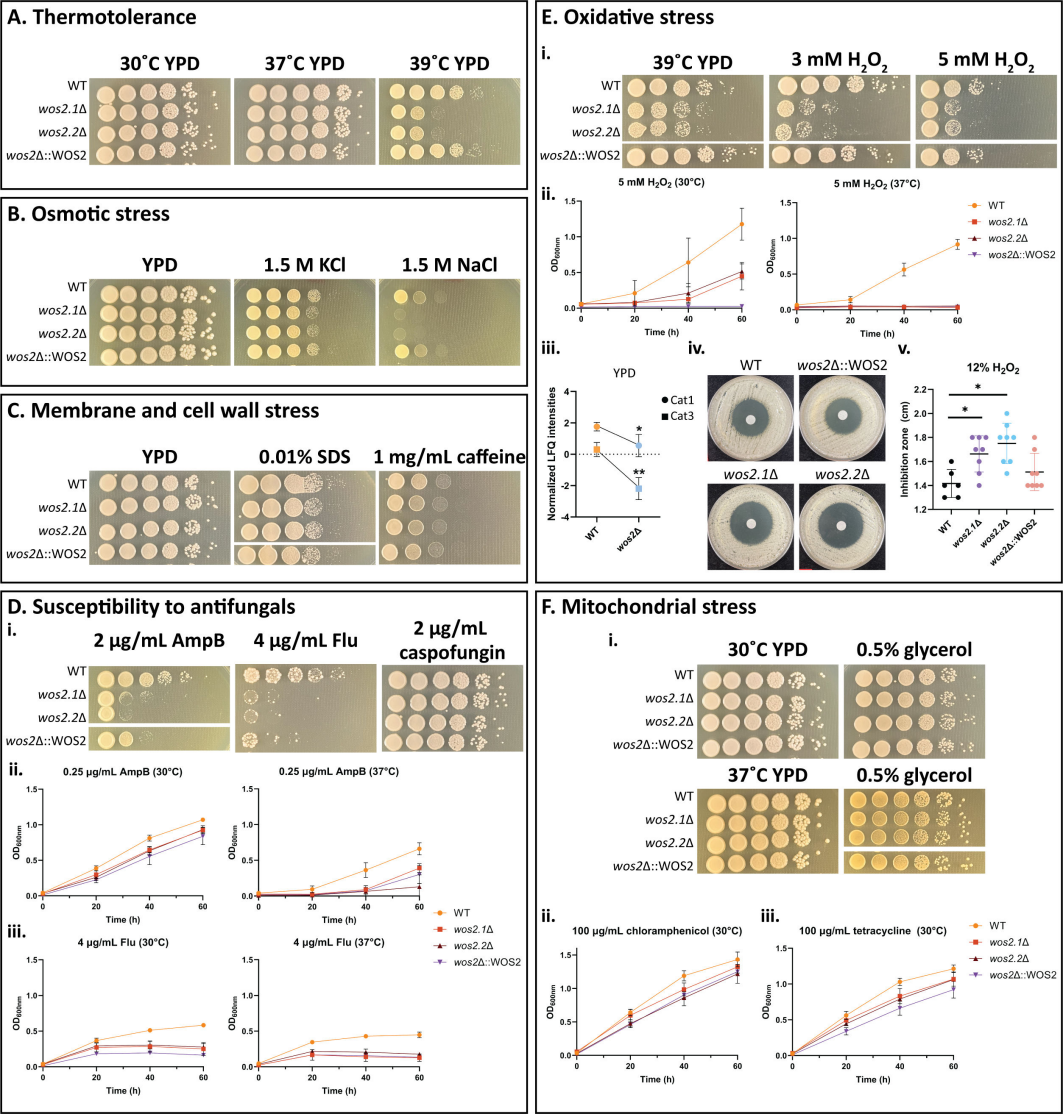
Wos2 influences fungal susceptibility to multiple stressors. (A-C) Phenotypic screening of *wos2*∆ mutant under A) rich conditions against increasing thermal stress (i.e., 30, 37, 39°C), (B) osmotic (i.e., 1.5 M NaCl, 1.5 M KCl), and (C) cell wall and membrane (i.e., 0.01% SDS, 1 mg/mL caffeine) stressors. (D) Phenotypic screening of *wos2*∆ to assess susceptibility to antifungal drugs. i) Stress dilution plate assays supplemented with 2 µg/mL amphotericin B (AmpB), 4 µg/mL fluconazole (Flu), and 2 µg/mL caspofungin. (ii-iii) Growth profile of *wos2*∆ strain in the presence of 0.25 µg/mL AmpB (ii) and 4 µg/mL Flu (iii) at 30 and 37°C. (E) Phenotypic screening of *wos2*∆ mutant against oxidative stress. (i) Stress dilution plate assays supplemented with 3 and 5 mM H_2_O_2_ at 39 ˚C. (ii) Growth profile of *wos2*∆ strain in the presence of 5 mM H_2_O_2_ at 30 and 37°C. (iii) Normalized mean label-free quantitative (LFQ) intensities for *C. neoformans* Cat1 and Cat3 proteins identified by proteomic profiling of YPD-enriched conditions. Experiment completed in biological quadruplicate. (iv) H_2_O_2_ disc diffusion across WT, *wos2*∆, and *wos2*∆::WOS2 strains in the presence of 12% H_2_O_2_. Experiments performed in biological quadruplicate and technical duplicate. (v) Oxidative stress sensitivity assessed from the H_2_O_2_ disc diffusion assay and measured based on the zone of inhibitions. 12% H_2_O_2_ is equivalent to 3.9 M. (F) Phenotypic screening of *wos2*∆ mutant against mitochondrial stressors. (i) Stress dilution plate assays supplemented with 0.5% glycerol at 30 and 37°C. (ii-iii) Growth profile of *wos2*∆ strain in the presence of 100 µg/mL chloramphenicol (ii) and 100 µg/mL tetracycline (iii) at 30°C. Serial dilutions of strains were spotted onto YPD and YPD supplemented with stressor and incubated at 30 and 37°C for 2-5 days, unless otherwise stated. For growth curve assays, strains were grown overnight in YPD and diluted to an OD_600nm_ of 0.1 in YPD and YPD supplemented with stressor and incubated at 30 or 37°C with OD_600nm_ measurements recorded. Experiment completed in biological triplicate and technical duplicate. Statistical analysis using Students *t* test: *, *P* < 0.05; **, *P* ≤ 0.001. Representative images of colony dilutions all originated from the same plate for each respective condition.

Fungal pathogens require robust adaptation strategies to endure stressors within hostile environments (e.g., during infection of a human host). A lack of homeostatic response can lead to cellular dysregulation in intracellular protein transport, disruption of cellular organization, and lethal proteotoxicity (8, 39). Given the homology of Wos2 to an Hsp90 co-chaperone and its important roles in proteomic reprogramming during both enriched and infection-mimicking conditions, we aimed to establish the requirement of Wos2 for fungal stress response. First, fungal growth on YPD at 30, 37, and 39°C was assessed to determine the role of Wos2 in fungal adaptation to increasing thermal stress and showed no difference among WT, *wos2*∆, and *wos2*∆::WOS2 for lower temperatures, whereas heat shock (39°C) sensitivity of *wos2*∆ revealed a dramatic decrease in fungal growth on YPD at elevated temperatures (Fig. 4A; Fig. S1). Next, we assessed the sensitivity of the *wos2*∆ mutant to osmotic stressors. We observed that fungal growth on multiple osmotic stressors, including NaCl and KCl, revealed a more pronounced growth sensitivity and a subtle growth impairment, respectively, upon exposure compared to WT (Fig. 4B). For assessment of cell membrane stress, *wos2*∆ featured a modest growth impairment compared to WT in the presence of the cell membrane stressor, SDS, and minimal differences in fungal growth in the presence of the cell wall stressor caffeine, an effector of signal transduction and cell wall integrity (Fig. 4C) (40).

Considering a prior study linking Wos2 with antifungal susceptibility in *N. cassa* (22), we assessed the susceptibility of the *wos2*Δ strain to cell wall and membrane targeting antifungals (Fig. 4D). We observed pronounced susceptibility to fluconazole and amphotericin B, antifungal drugs that target the fungal plasma membrane, and no differences in fungal growth to caspofungin, a cell wall targeting antifungal drug (41). Next, we corroborated our observed *wos2*Δ sensitivities to amphotericin B by profiling changes in fungal growth during amphotericin B stress and detected increased susceptibility of *wos2*Δ at higher temperatures. The growth profile of *wos2*Δ was also impaired in the presence of fluconazole stress at 30 and 37°C with consistent reduction in growth in the absence of *wos2* across temperatures, with a significant increase in susceptibility at 1.0 µg/mL fluconazole in a limiting dilution assay (Fig. S2A through B). The phenotypic divergences were restored in *wos2*∆::WOS2 to WT levels in a fluconazole agar assay (Fig. S2C) and partial restoration of amphotericin B sensitivity across increasing drug concentrations and multiple temperatures (Fig. S2D), verifying the involvement of Wos2 in antifungal drug adaptation.

Given our functional enrichment observations of *wos2*Δ in processes that indicate a lack of stress-responsive translational remodeling (i.e., ribosome and translation elongation factor activity; Fig. 3D and E) combined with our observations of WT-abundant oxidative stress factors (i.e., catalases and peroxin), we evaluated the impact of oxidative stress on the *wos2*∆ strain. As anticipated, growth of the fungal strains in the presence of 5 mM H_2_O_2_ (hydrogen peroxide, an oxidative stressor) showed a reduction in growth for *wos2*∆ vs. WT (Fig. 4E). As anticipated, growth of the fungal strains in the presence of 3 mM H_2_O_2_ (hydrogen peroxide, an oxidative stressor) showed a reduction in growth for *wos2*∆ vs. WT, with elevated sensitivities observed upon increased concentration (i.e., 5 mM H_2_O_2_). We also profiled changes in fungal growth of *wos2*Δ in the presence of 5 mM H_2_O_2_ and observed impairment at 30°C; this susceptibility became more pronounced upon increased temperature stress (i.e., 37°C) . The *wos2*Δ oxidative susceptibility was completely restored in *wos2*∆::WOS2 to WT levels in elevated stress conditions (i.e., 5 mM H_2_O_2_, 39°C), confirming the requirement of Wos2 for adaptation to multiple stressors . Further, it is recognized that single and quadruple mutant strains (i.e., *cat1, cat2, cat3,* and *cat4*) of the catalase family in *C. neoformans* have not previously exhibited an oxidative stress phenotype during elevated levels of exogenous or endogenous stress (27); however, catalase activity is one arm of a multipronged fungal antioxidant system, which may have interchangeable roles with other undiscovered constituents. Excitingly, proteomics profiling data defined a significant reduction in catalase (Cat11 and Cat3) production in the *wos2*∆ vs WT strains , which sustain increased ROS protection for the WT strain and a quantitative assessment of the zone of inhibition measurements revealed a significant growth sensitivity in the *wos2*Δ strain . On the basis of previous reports correlating mitochondrial contributions to oxidative and antifungal stress resistance (42–44), we examined the susceptibility of *wos2*Δ on the alternative carbon source glycerol, which is metabolized via mitochondria-dependent processes, and revealed minor mutant impairment (Fig. 4F). Additionally, investigation of drugs targeting mitochondrial function, including tetracycline and chloramphenicol, did not differentially affect the growth of *wos2*Δ. Collectively, these results support Wos2 as a novel mediator of fungal adaptation to ROS.

### Secretome profiling further elaborates on the role of Wos2 in oxidative stress response

**Fig 5:**
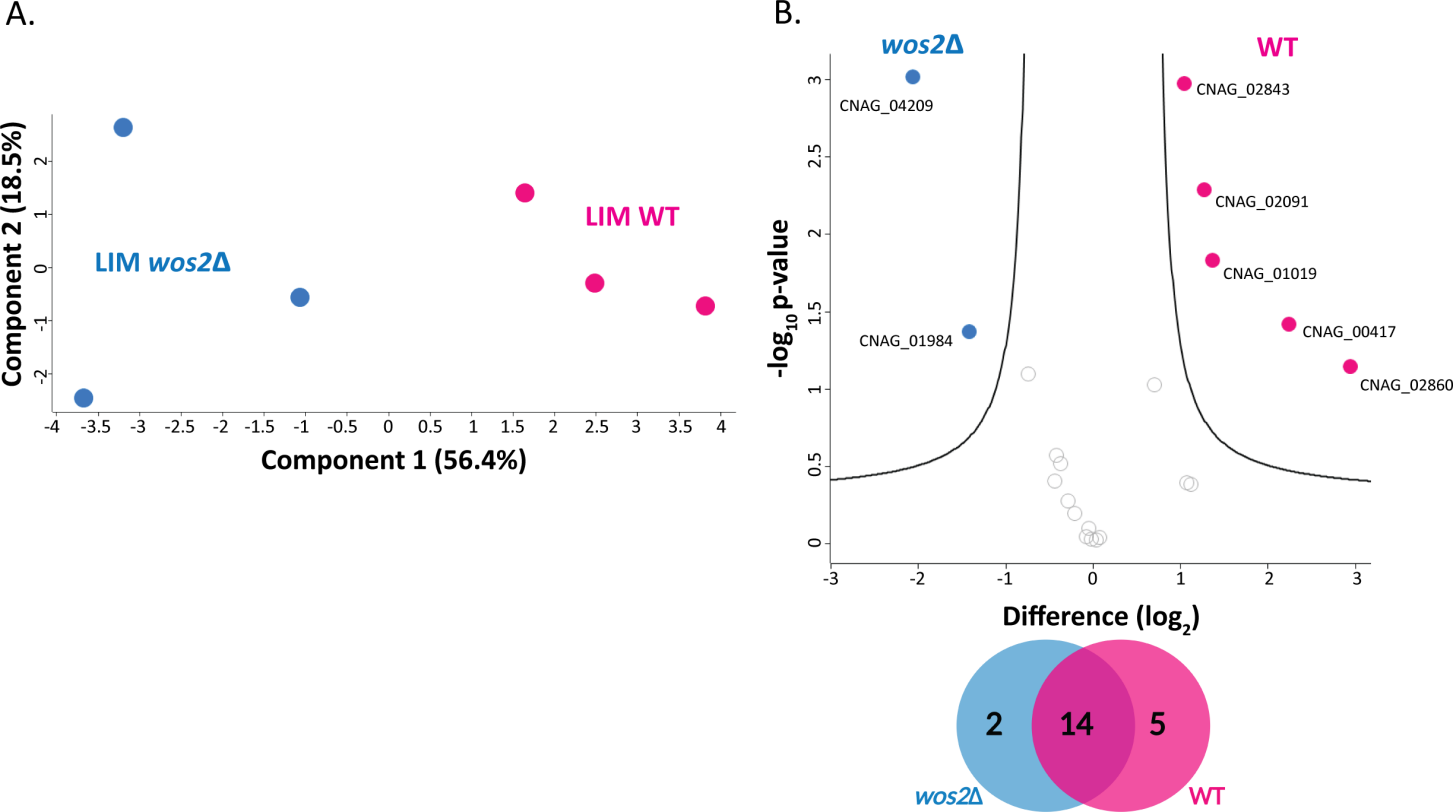
Secretome profiling of Wos2 in infection-mimicking conditions. (**A**) Principal component analysis of the experiment profiled in LIM (infection-mimicking) conditions between WT and *wos2*∆ strains. (**B**) Volcano plot comparing all proteins identified under infection-mimicking (i.e., LIM) conditions between WT and *wos2*∆. Highlighted proteins depict significant changes in protein abundance upon strain comparison; Venn diagram depicts the number of significantly different proteins identified in each condition. Student’s *t* test, *P* < 0.05; FDR = 0.05; S_0_ = 1. The experiment was performed in biological triplicate.

The extracellular environment serves as an important interaction point for fungal pathogens to respond to the milieu of host-generated ROS as a first line of defense and subsequent adaptation (3). Given the pronounced Wos2-associated signature within the infection-mimicking cellular proteome, we explored if Wos2 exhibits similar control over the extracellular environment in host-like conditions. In this study, we profiled the secretome across WT and *wos2*∆ strains when grown in LIM. A PCA plot indicated the largest component of separation was deletion of *wos2* (component 1, 56.4%), with a second component of distinction defined by biological variability (component 2, 18.5%) (Fig. 5A). Comparison by volcano plot revealed distinct Wos2 signatures of the fungal extracellular environment under infection-mimicking conditions, with five proteins significantly more abundant in WT vs two proteins significantly more abundant in the deletion strain (Fig. 5B; Table S2). For the WT, we observed a significant increase in abundance of the well-characterized *C. neoformans* virulence factor, superoxide dismutase (SOD1, CNAG_01019; >1.3 fold [log_2_]), a protein previously reported within fungal extracellular vesicles as critical for detoxifying oxygen radicals (11, 45). These data further support the role of Wos2 in the universal oxidative stress response. We also identified endo-1,3(4)-β-glucanase (CNAG_02860; >2.9 fold [log_2_]), a crucial hydrolyzing enzyme putatively involved in capsule attachment and cell wall remodeling (46), corroborating the observed loss of membrane integrity (i.e., SDS, amphotericin B, and fluconazole stress, Fig. 4C and D ) as well as our previous observations of significantly reduced capsule:cell ratio of *wos2*Δ (3). In addition, an uncharacterized protein (CNAG_02843; >1.0 fold [log_2_]), a nucleosome assembly protein (CNAG_02091; >1.2 fold [log_2_]), and elongation factor 1-gamma (CNAG_00417; >2.2 fold [log_2_]) were significantly more abundant in WT. Conversely, we identified a voltage-gated potassium channel subunit (CNAG_04209; >2.0 fold [log_2_]) with predicted importance in regulating membrane potential (47), as well as a previously reported moonlighting protein, transaldolase (CNAG_01984; >1.4 fold [log_2_]) (48), to be more abundant in the *wos2*∆ strain. Altogether, these results support the role of Wos2 in modulating the secretory profile of *C. neoformans* under nutrient limiting conditions for preparation against host defenses, including ROS.

### Wos2 mediates intracellular fungal survival and replication within macrophages and attenuates fungal virulence

**Fig 6:**
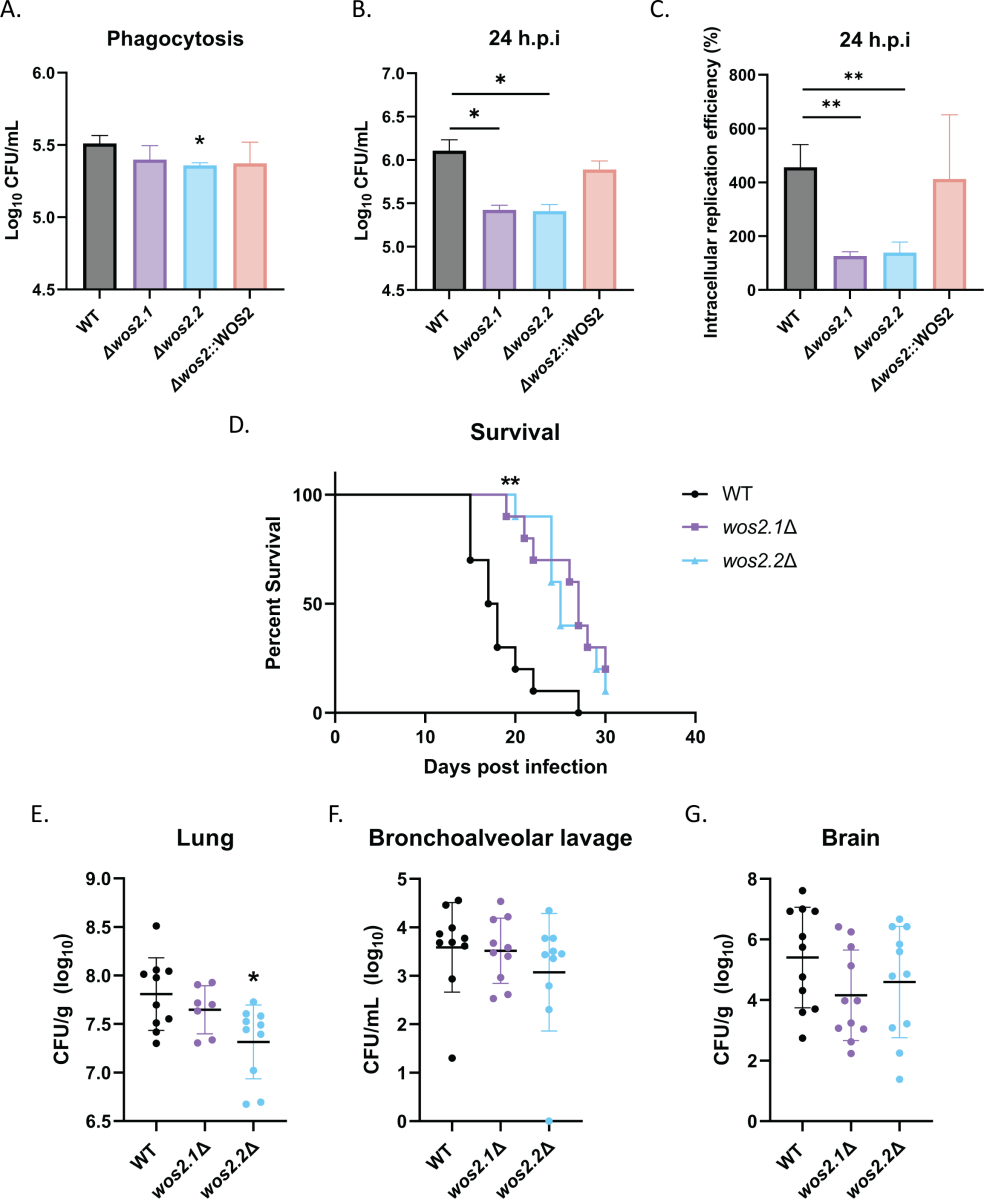
Characterization of Wos2 during *in vitro* and *in vivo* infection assays. (**A**) Phagocytosed fungal cells were quantified by co-culturing macrophages with WT and *wos2*∆ strains for 3 hours. Infected cells were washed six times with PBS, lysed, and plated for CFUs. (**B**) Fungal burden was assessed following initial 3-hour co-culture and washing six times in PBS to remove extracellular and non-adhered fungal cells, followed by maintenance of infected macrophages in Dulbecco's modified Eagle medium (DMEM) supplemented with 20 µg/mL fluconazole. At 24 h.p.i, host cells were lysed and plated for CFUs. (**C**) Intracellular replication efficiencies were calculated by quantifying the number of CFUs in the supernatant and host cell at 24 h.p.i. Statistical analysis was done using Student’s *t-*test: *, *P* < 0.01; **, *P* ≤ 0.005. Experiments were completed in biological triplicate and technical duplicate. (**D**) BALB/c mice infected with *C. neoformans* WT and *wos2*∆ succumbed to infection, or *wos2*∆ mutant survived to the assay endpoint (i.e., 30 days). Differences in survival were statistically tested using a log-rank (Mantel–Cox) test (**, *P* ≤ 0.001). (**E–G**) Fungal burden from lung (**E**), bronchoalveolar lavage fluid (**F**), and brain (**G**) determined measuring CFUs. Statistical analysis using Student’s *t*-test (*, *P* < 0.01).

Given the role of Wos2 in modulating the cryptococcal response to ROS, we predicted that Wos2 has a role in fungal infection. First, we confirmed production of Wos2 in *C. neoformans* in the presence of macrophages through immuno-microscopy with cytosolic localization (Fig. S3) (4). Next, given our defined role for Wos2 in oxidative and membrane stress and fungal virulence and the ability of *C. neoformans* to interact and survive within macrophages, especially within the harsh environment of the phagosome (e.g., ROS, reactive nitrogen species, nutrient starvation conditions, and acidic pH) (49), we speculated that *wos2*∆ strains would be compromised in ROS detoxification abilities and demonstrate increased sensitivity within the phagosome environment. To test our hypothesis, we first assessed the susceptibility of the strains to fungal killing by macrophages at an early infection timepoint via co-culturing the fungal strain with immortalized BALB/c macrophages. We observed a slight reduction in intracellular burden levels for the *wos2*∆ strains (significant reduction for one independent mutant), suggesting Wos2 may influence the initial interactions with macrophages, but likely not to biologically relevant levels (Fig. 6A). Notably, our previous observations of enlarged *wos2*∆ cell size may support such impeded fungal uptake by macrophages (3). We then assessed the importance of Wos2 within a prolonged infectious state by quantifying intracellular populations of *C. neoformans* upon macrophage exposure. At 24 hours post-infection (h.p.i), we observed a significant reduction in fungal burden for the *wos2*∆ strains relative to WT (Fig. 6B). To address whether these fungal survival variances were associated with differing initial macrophage–fungal interactions or intracellular replication, we applied a fluconazole protection assay at 24 h.p.i. (50). Assessment of CFU counts from the supernatant and lysed macrophages at 24 h.p.i. normalized to CFUs of the phagocytosed fungal strain at 3 h.p.i identified a significant impairment in replication efficiencies for *wos2*∆ compared to WT (Fig. 6C). These results are consistent with our fungal killing observations at 24 h.p.i., but we acknowledge that further reduced levels of *wos2*∆ may be influenced by increased susceptibility to fluconazole. Critically, these data align with our *in vitro* phenotypic assay findings and proteomic results to support a deficit for *wos2*∆ to replicate under ROS-inducing conditions.

Given the *wos2*∆ virulence defect in oxidative stress and impaired affinity for the intracellular macrophage environment, we predicted that *wos2*∆ would display reduced virulence in a murine inhalation model for cryptococcosis. Following inoculation, we observed a significant increase in murine survival with the *wos2*∆ strain compared to WT (Fig. 6D). Moreover, to assess if differences in survival were attributed to reduced fungal burden, we quantified a significant decrease of fungal cells within the lungs infected with one independent mutant (*wos2.2*∆) (Fig. 6E). Notably, the second independent mutant (*wos2.1∆*) showed the same trend in reduced fungal burden, but not to a significant level. Bronchoalveolar lavage did not reveal any significant changes in fungal burden compared to WT (Fig. 6F), as also noted within the brain (Fig. 6G), although a reduction in fungal counts for both organs was detectable. The attenuated virulence of *wos2*Δ was confirmed by the reconstituted strain partially restoring virulence in a murine survival assay determined with a comparable survival curve (*P* = 0.3225) and consistent fungal burden levels within the lungs, bronchoalveolar lavage, and brain compared to WT (Fig. S4A through D). The virulence stability of the WT strain was confirmed between experiments by comparison of survival curves (i.e., 17.5 days vs 18.5 days median survival; *P*-value = 0.2099). Taken together, these data support Wos2 as an important contributor to *C. neoformans* virulence with an impaired ability to cause disease in an *in vivo* model of cryptococcosis without concretely impacting the fungal burden across the lungs and brain.

## DISCUSSION

A promising approach to combatting the current limited antifungal supply is to generate novel therapeutics that interrupt core fungal stress responses, such as the Hsp90 chaperone. However, due to close evolutionary history, overcoming host toxicity is a significant hurdle (7, 18, 51). Therefore, elucidating the roles of Wos2 and other co-chaperones offers a novel resource for drug development and understanding foundational stress responses in fungal pathogens. For instance, hypersensitivity to the Hsp90 inhibitors, geldanamycin and radicicol, was observed in yeast lacking the Wos2 homolog, p23 (52). Furthermore, Hsp90 co-chaperones may feature stress-specific contributions depending on the type of the stressor; for example, p23 deletion in the model filamentous organism, *N. cassa,* resulted in hypersensitivity to azoles and heat, whereas no susceptibility to amphotericin B or H_2_O_2_ was detected (22). In comparison, we demonstrate coinciding *wos2* specific heat and fluconazole sensitivities and pronounced susceptibility to peroxide and amphotericin B stressors. This contrasting Wos2/p23-stress signature may be attributed to the pathogenic lifestyle of *C. neoformans,* requiring robust and redundant stress responses to survive within a host. Furthermore, the observed *wos2*∆*-*specific stress sensitivities are characteristic of an impaired Hsp90 co-chaperone network and complement our observed enrichment of GOBP terms associated with translation and protein folding upon *wos2* deletion. Specifically, our proteome characterization of *wos2*Δ in infection-like stress identified a plethora of co-chaperones important for protein folding and temperature stress with significantly increased abundance, such as Ydj1p/Hsp40 (CNAG_03944), Hsp10 (CNAG_03982), CCTβ (CNAG_00447), and DnaJ (CNAG_05252) (53–56). Altogether, we suggest that the deletion of Wos2 results in aggravated cellular dysbiosis, requiring reliance on numerous protein folding mechanisms to rescue cellular homeostasis.

Common sources of oxidative stress generation in human fungal pathogens during infection are generated from elevated mammalian body temperatures (37°C–39°C) and exposure to antifungal drugs, including azoles and polyenes (57–60). Thus, our observations of Wos2 requirement for resistance to membrane targeting antifungal drugs, heat, and peroxide stress support a common theme of accumulated intracellular ROS from the deletion of *wos2*. Moreover, cell wall stress generated by caffeine and caspofungin did not result in elevated susceptibility in *wos2*∆, proposing that the aforementioned sensitivities are not attributed to decreased cell wall integrity (5, 13). Given that Hsp90 mediates fluconazole efficacy in *C. neoformans* (5)*,* our observed dramatic affluence of fluconazole stress on *wos2*∆, and a previous report highlighting the accumulation of azole-derived toxic intermediates in a *p23* mutant of *N. cassa,* it would be of interest to investigate if the contributions of Wos2 to basal azole resistance is due to ergosterol stability, detoxification of ROS, or elimination of toxic intermediates (22). Interestingly, we observed a significant increase in a key protein in ergosterol synthesis, CNAG_03819 (*ERG6*), in *wos2*∆ under infection-mimicking conditions, supporting the involvement of Wos2 in ergosterol production (61). However, as this analysis did not investigate the Wos2 proteome response to azole stress, we cannot concretely correlate Wos2-induced azole susceptibility to ergosterol biosynthesis.

The ability to cause and maintain infection within a host depends on the capacity to endure the released extracellular ROS milieu and the oxidative burst within host immune cells. High levels of oxidative stressors result in catastrophic disruptions in homeostatic cellular functions via oxidation of proteins, lipids, and nucleic acids (62). Thus, fungal pathogens evolved multipronged antioxidant protection initiated with translational prioritization to rapidly direct translational machinery to prioritize stress-related mRNAs over homeostatic mRNAs (35, 63–65). This phenomenon occurs in *C. neoformans* for conditions apart from oxidative-related stress, including nutrient starvation and heat shock (34–36, 66). Simultaneously, stress-induced remodeling governs the production of stress-response factors, including chaperoning networks, to repair and prevent cellular damage, ensuing multiple safety precautions to maintain fungal survival (36, 67). We observed infection-mimicking conditions induced enrichment of non-stress-related processes within the *wos2*Δ proteome (i.e., translation, amino acid biosynthesis, and nucleotide metabolism) with decreased responsiveness in major antioxidant enzymes (i.e., Sod1, Cat1, and Cat3), corroborated by an oxidative sensitivity phenotype (i.e., H_2_O_2_). Our results suggest that Wos2 is a critical intermediary during nutrient limitation and oxidative stress, vital for promoting a stress-resistant proteome through translational regulation and production of stress-response proteins. We have yet to elucidate the role of Wos2 within the stress adaptation pathway, i.e., whether Wos2 is vital for sensing or supporting the global response to exogenous and endogenous stress. Both of these factors are an arm within the Hsp90 chaperone network (4, 5, 18, 68). Specifically, *wos2* deletion resulted in a significant abundance of translation initiation factor proteins (i.e., eIF2α and eIF2β) and a substantial increase in ribosomal proteins (11.2% of *wos2*∆-significantly produced proteins) within the infection-mimicking condition, consistent with previous reports highlighting p23 regulation of ribosomal biogenesis (69). This is of importance due to reports indicating that *C. neoformans* growth in oxidative and nutrient-limiting stress results in severely reduced global translation by eIF2α inhibition, preventing the assembly of the pre-ribosomal complex, resulting in preferable accumulation of stress-responsive mRNAs instead of energetically expensive mRNAs, such as ribosomal proteins (34, 36, 66, 70).

The observed dysregulation within the *wos2*∆ proteome is evidenced by the Wos2 implications in cellular stressors (i.e., oxidative, membrane, osmotic, and temperature), suggesting Wos2 elicits protection and adaptation in an infectious environment. Moreover, our previous characterization of the influence of Wos2 on classical virulence attributes, including a decrease in capsule production, decrease in thermotolerance in nutrient-altered conditions, and altered melanin pigmentation, further supports the dependency on Wos2 for fungal adaptation (3). Thus, the impaired macrophage intracellular replication levels of *wos2*∆ arose from an inability to obtain a satisfactory replication niche, consequently reducing affinity to the phagosomal environment. Multiple lines of evidence support this statement as our *in vitro* stress assays may be translated to different facets of the fungicidal nature of the phagosome, including Wos2 requirements for osmotic (i.e., low pH and high cation concentration in matured phagosome) and oxidative (i.e., phagolysomal oxidative burst) adaptation, as well as the necessary Wos2 support for capsule enlargement to confer resistance to the phagosome (45, 71, 72). However, the observed *wos2*∆ dysregulation is not absolute, as the mutant strains partially attenuated virulence in a murine model. Thus, Wos2 is an important component of establishing fungal virulence; however, it is not a requirement to cause disease. It is known that *C. neoformans* contains a redundant and functionally overlapping assortment of virulence and protection mechanisms; however, how these responses to host-derived cellular stressors are produced is not frequently addressed. Thus, this study supports the idea that the fungal adaptation network contains preliminary “fail-safes” apart from virulence factors that ensure pathogen durability. Overall, this study highlights the delicate host–fungal interaction balance and how debilitating a significant arm of the fungal stress response network leads to compensatory proteome regulation to maintain control and infection within the host.

Finally, a critical limitation of this study is the partial restoration of Wos2 to WT levels in *wos2*∆::WOS2. In contrast, we observe almost complete restoration of *wos2*∆ susceptibility in the complement strain across the investigated stressors and the virulence assays; however, in liquid growth assays, we observe similar susceptibility between mutant and complement strains. A possible explanation for this variable restoration is the interrupted functioning of our recombinant Wos2 due to the addition of a Flag-tagged C-terminus domain, whose intended use enabled immunofluorescence. Reports have highlighted vital elements of the disordered tail within the C-terminal domain of p23. Specifically, the unstructured tail of p23 is pivotal for coordinating client binding and progression of the Hsp90 chaperone cycle by guiding the client to Hsp90 and stabilizing the Hsp90–client complex (17, 73, 74). In this context, the long unstructured tail features regions of varying flexibility and helical propensity; these motifs allow engagement to a wide spectrum of client proteins (17). Thus, our addition of an acidic Flag tag does not interfere with protein production (3); however, it could be detrimental to protein functioning in specific scenarios. It is possible that liquid-based methods increase chemical exposure to the cell (i.e., chemical exposure restricted to the surface on agar plate), resulting in an increase in client-binding requirements that the impaired Wos2-Flag is unable to manage (75). Nonetheless, appropriate restoration of Wos2A must be conducted to resolutely link Wos2 to oxidative adaptation.

### Conclusions

Chaperones are a critical line of fungal protection against cellular proteotoxic damage induced by high temperatures and environmental stressors. This global stress response comprises overlapping chaperone and co-chaperone networks consisting of many clinically relevant proteins essential for multiple facets of fungal disease. Partitioning putative co-chaperones, such as Wos2, as a potential therapeutic avenue facilitates the inhibition of a layer of Hsp90 regulation and the entire co-chaperone stress response system. Therefore, it is important to detail the involvement of a co-chaperone in both fungal virulence and fitness, as the ability of a fungal pathogen to cause disease is fitness-conditional. Our study provides a detailed investigation into the co-chaperone Wos2 and defines distinct Wos2-controlled fitness and virulence attributes vital for response to environmental threats and establishing fungal infection.

## MATERIALS AND METHODS

### Fungal strains, growth conditions, and media

*Cryptococcus neoformans* var. *grubii* wild-type (WT) strain H99 (serotype A) was used for all analyses and as a reference strain for mutant construction. *C. neoformans* mutant (*wos2*Δ) and complement strains (*wos2*Δ::WOS2) were constructed using biolistic transformation of constructs amplified using double-joint PCR or Gibson assembly (NEB), as previously described (primers, strains, and plasmids provided) (3). Two independent mutant strains were constructed for *wos2;* two reconstituted strains were constructed for *in vitro* (restored eposomally, *wos2-4x FLAG::HYG-pHP1*) and *in vivo* (re-integrated into genome, *wos2-1xFLAG::HYG*) experimental assays (strains listed in S3). The wild-type strain was maintained on yeast peptone dextrose (YPD) agar plates (2% dextrose, 2% peptone, 1% yeast extract, and 1% agar), and mutant and complement strains were maintained on YPD supplemented with 100 µg/mL nourseothricin (NAT) and 200 µg/mL hygromycin B, respectively, at 30°C, unless otherwise stated.

For YPD (i.e., enriched) proteomic sample collection, *C. neoformans* strains were inoculated in YPD media overnight at 37°C, followed by subculture into fresh YPD and grown to mid-log phase (approx. 13 hours). For infection-mimicking proteomic sample collection, fungal strains were inoculated in YPD media overnight at 37°C, followed by subculture in the yeast nitrogen base (YNB) medium with amino acids (BD Difco, Franklin Lakes, NJ) supplemented with 0.05% dextrose overnight. Samples were collected, washed in low iron capsule-inducing media (CIM) and sub-cultured in CIM to mid-log phase (approx. 37 hours) (23). Proteomic samples were collected in biological quadruplicate.

### Proteomics sample preparation

Samples for mass spectrometry were prepared as previously described (76). Briefly, samples were collected and washed twice in 1 x phosphate-buffered saline (PBS) and resuspended in 100 mM Tris-HCl (pH 8.5) containing a protease inhibitor cocktail tablet (Roche). Following the addition of sodium dodecyl sulfate (SDS, 2% final concentration), samples were lysed using a probe sonicator (Thermo Fisher Scientific). Dithiothreitol (DTT, 10 mM final concentration) was added, and samples were incubated at 95°C with 800 rpm agitation for 10 minutes, followed by incubation with iodoacetamide (IAA, 55 mM final concentration) for 20 minutes in the dark. Samples were acetone-precipitated (80% acetone final concentration) overnight at −20°C, then collected and washed twice in 80% acetone, and resuspended in 8 M urea/40 mM HEPES for protein quantification using a bovine serum albumin (BSA) tryptophan assay (77). Samples were diluted in 50 mM ammonium bicarbonate and normalized to 50 µg protein for overnight LysC/trypsin digestion (Promega, protein:enzyme ratio, 50:1). Trifluoroacetic acid (TFA, 10% v/v) was added to quench the digestion, and peptides were purified using C18 Stop And Go Extraction (STAGE) tips (78).

Secretome sample preparation was performed using an in-solution digestion as previously described (76). Briefly, the culture supernatant was filtered to remove whole cells and cellular debris by 0.22-µm syringe filters and incubated at 95°C for 10 minutes, followed by the addition of one-third volume of 8 M urea/40 mM HEPES to the filtered sample. Samples were ultrasonicated in an ice bath (15 cycles, 30 seconds on/30 seconds off) and then reduced and alkylated with DTT and IAA. Samples were then enzymatically digested overnight, and STAGE-tip-purified.

### Liquid chromatography–tandem mass spectrometry

Liquid chromatography–tandem mass spectrometry was performed as previously described with some modifications (28). Lyophilized peptides were resuspended in buffer A (0.1% formic acid) and analyzed on an Orbitrap Exploris 240 hybrid quadrupole-orbitrap mass spectrometry (Thermo Fisher Scientific) coupled to an Easy-nLC 1200 high-performance liquid chromatography device (Thermo Fisher Scientific). Resuspended samples were first loaded and separated on an in-line PepMap RSLC EASY-Spray column (75 µm by 50 cm) filled with C_18_ reverse-phase silica beads (2 µm) (Thermo Fisher Scientific). Peptides were subsequently electrosprayed into the mass spectrometer instrument across a linear gradient of 0%–32% buffer B (80% acetonitrile, 0.5% acetic acid) over a 110-minute gradient, followed by washing in 95% buffer B for 5 minutes, and held for 5 minutes with 4% buffer B, with a 250 nL/min flow rate. The mass spectrometer cycled between one full scan and MS/MS scans of the Top10 abundant peaks. Full scans (*m/z* 400 to 1,600) were captured in the Orbitrap mass analyzer with a resolution of 60,000 at 200 *m/z*.

### Data processing

Data analysis of the mass spectrometry .RAW data files was completed using MaxQuant software (version 2.1.3) (25). The search was performed using the integrated Andromeda search engine against the reference *C. neoformans* var. *grubii* serotype A (strain H99/ATCC 208821) proteome (7,429 sequences; downloaded on 14 July 2022) from Uniprot (79). The following parameters were included for data processing: trypsin enzyme specificity with maximum two missed cleavages; minimum peptide length of seven amino acids; fixed modifications—carbamidomethylation of cysteine; variable modifications—methionine oxidation and N-acetylation of proteins. Peptide spectral matches were filtered with a target-decoy approach at a fals -discovery rate (FDR) of 1% with a minimum of two peptides required for protein identification. Relative label-free quantification (LFQ) and match between runs were enabled, and the MaxLFQ algorithm used a minimum ratio count of 1 (80).

### Bioinformatics

Statistical analysis and data visualization were completed using Perseus (version 1.6.14) (26). Data were filtered for reverse database hits, contaminants, and proteins only identified by site. LFQ intensities were log_2_-transformed and filtered for valid values (three of four replicates in at least one group), followed by imputation of missing values from the normal distribution (width, 0.3; downshift, 1.8 standard deviations). Significant differences were evaluated by Student’s *t-*test (*P* ≤ 0.05) with multiple-hypothesis testing correction using the Benjamini–Hochberg FDR at 0.05 with S_0_ = 1 (81). A 1D annotation enrichment (i.e., tests for each annotation term whether the respective numerical values have a preference to be larger or smaller than the global distribution of the values for all proteins) was performed based on GOBP terms described with an FDR threshold of 0.05 using the Benjamini–Hochberg method (38). Visualization of protein networks was performed using STRING analysis as described at https://string-db.org/ (37). Protein–protein interaction networks were generated with STRING basic settings and medium confidence interval (i.e., 0.4). STRING enrichment analysis was completed in the statistical background of the whole genome of *Cryptococcus neoformans* var. *neoformans* JEC21; local network cluster (STRING) enriched functional clusters with an FDR threshold of 0.05 and *P* ≤ 0.05.

### Disk diffusion assay

The susceptibility of the *C. neoformans* strains to oxidative stress was assessed as previously described (27, 28). *C. neoformans* strains were grown to mid-log phase in YPD at 37 ˚C; 2.5 × 10^5^ cells were plated with a cotton swab on semi-solid YPD. Sterile filter discs (Whatman MM, 10 mm diameter) were placed in the plate center, and 15 µL of 12% H_2_O_2_ was added to the disc. Plates were incubated at 37°C for 48 hours, photographed, and measurements were taken from three locations to the nearest millimeter to determine the radius of the zone of inhibition. The experiment was completed with four biological replicates and in technical duplicate.

### Cell culture

BALB/c WT immortalized macrophages (generously provided by Dr. Felix Meissner, Max Planck Institute of Biochemistry, Germany) were maintained at 37°C in 5% CO_2_ in Dulbecco’s modified Eagle’s Medium (DMEM) supplemented with 10% heat-inactivated fetal bovine serum (FBS; Thermo Fisher), 2 mM glutamax, 1% sodium pyruvate, 1% L-glutamine, and 5% pen/strep. For CFU assays, macrophages were seeded in 12-well plates at 0.1 × 10^6^ cells/well and grown until 70%–80% confluence (i.e., 0.5 × 10^6^ cells/well). For fluorescence microscopy assays, 0.3 × 10^6^ cells were seeded in 6-well plates and grown until 70%–80% confluence (i.e., 1.2 × 10^6^ cells/well).

### C. neoformans colony-forming unit counts and fluconazole protection assays

A *C. neoformans* infection and fluconazole protection assay was performed as previously described with modifications (50). Briefly, *C. neoformans* strains were grown to the mid-log phase in YPD at 30°C, collected, and washed twice in PBS. Macrophages were infected at a multiplicity of infection (MOI) of 5:1 (fungi:macrophage) in DMEM with pen/strep for 3 hours at 37°C at 5% CO_2_. Following co-culture, infected cells were extensively washed six times with PBS to remove any adhered or non-phagocytosed fungal cells, and fresh medium supplemented with 20 µg/mL fluconazole was added for the remainder of the assay. At the indicated timepoints (i.e., 12 and 24 hours), culture medium containing extracellular fungal cells was collected, and the infected macrophages were washed six times with PBS and lysed with 0.5% Tween-20 at room temperature for 10 minutes. The removed medium containing extracellular fungal cells was centrifuged at 1,200 x g for 12 minutes. The medium containing fluconazole was removed, and the cell pellet was resuspended in PBS. Serial dilutions of both resuspended extracellular fungal cells from culture media and lysed intracellular fungal cells were performed followed by plating on YPD and incubation for 48 hours at 30°C.

To measure the number of fungal cells engulfed at the starting timepoint, 3 h post-inoculation (h.p.i.), the cell lysis as described above was performed. The 12 and 24 h.p.i collection timepoints consisted of both extracellular and intracellular CFU assessments, as described above. The experiment was completed with three biological replicates and in technical duplicate.

To calculate fungal intracellular replication efficiency, for experiments that began at the initiation of infection, t_0_ (i.e., 3 hours) and advance to the indicated timepoints, t_n_ (i.e., 24 hours), the following formula was applied (50):


$$Intra.\;Rep.\;Efficiency:\left\{\frac{\left[\left(Extracelllular\;CFU\;at\;t_{n}\right)+\left(Intracellular\;CFU\;at\;t_{n}\right)\right]}{Intracellular\;CFU\;at\;t_{0}}\right\}\times \;100\%$$

### Dilution plate assay

To analyze *C. neoformans* Wos2 response to heat, oxidative, osmotic, and cell-wall stressors, dilution plate assays were performed as previously described (34, 40). To assess osmotic stressor phenotypes, NaCl (1.5 M) and KCl (1.5 M) were supplemented in the YPD medium. Cell wall and osmotic stressor phenotypes were assessed by adding caffeine (1 mg/mL), caspofungin (2 µg/mL), SDS (0.01%), amphotericin B (2 µg/mL), fluconazole (4 µg/mL), or H_2_O_2_ (3 mM and 5 mM) to the YPD medium. *C. neoformans* strains were grown to the mid-log phase in YPD at 30°C and serially diluted in tenfold (10^6^ cells/5 µL) on YPD plates supplemented with the different stressors and incubated at 30°C and 37°C, unless otherwise stated. Images were taken every 24 hours for 5 days. The experiment was completed in biological triplicate and technical duplicates.

### Growth curves

To analyze *C. neoformans* Wos2 response to stressors in a liquid broth assay, growth curves were performed as previously described with minor modifications (82, 83). Fungal cells were grown overnight in YPD at 30°C and diluted to an OD_600nm_ of 0.1 in 200 µL YPD or YPD supplemented with stressors in 96-well plates and incubated at either 30°C or 37°C. OD_600nm_ measurements were obtained on BioTek HM1 plate reader every 15 minutes over 70 hours. Oxidative, membrane, and mitochondrial stressors were added to the YPD medium, including fluconazole (4 µg/mL), amphotericin B (0.25 µg/mL), H_2_O_2_ (5 mM), chloramphenicol (100 µg/mL), and tetracycline (100 µg/mL). Each assay was completed in biological triplicate and technical duplicate.

### Limiting dilution antifungal sensitivity assay

Assessment of the MICs required to inhibit growth of *wos2*∆ relative to that of untreated cells was done as described previously with slight modifications (41). Briefly, cells were grown overnight in YPD at 30°C and diluted to an OD_600nm_ of 0.01 in YNB. Sensitivity to fluconazole was determined using concentrations over a five-dilution series of 0.48 to 7.6 µg/mL. Treated and control cells were statically incubated in clear, round-bottom 96-well plates at 30°C for 48 hours, and the OD_600nm_ was measured. The experiment was completed in biological quadruplicate and technical duplicate. Data were reported as the percentage of cell density relative to that of untreated cells per respective strain.

### Immunofluorescence

BALB/c WT immortalized macrophages were maintained as described above; to minimize autofluorescence, cells were grown in FluoroBrite DMEM media (Thermo Fisher Scientific) (supplemented with 10% FBS and 1% L-glutamine) 24 hours prior to infection and throughout the infection protocol. Macrophages were infected at an MOI of 100 for 3 hours at 37°C at 5% CO_2_, and samples were collected following co-culture and washing twice with PBS. The protocol for immunostaining was adapted from previously described methods (84–86). Briefly, cells were fixed overnight in 4% paraformaldehyde in PBS at 4°C and plated onto 0.1% poly-L-lysine (Sigma-Aldrich)-coated cell slide. Cells were incubated with buffer (0.1 M sodium citrate; 1.1 M sorbitol pH 5.5) containing 10 mg/mL of lytic enzymes (Sigma, L1412) and a protease inhibitor cocktail tablet and incubated at 30°C for 2 hours. Slides were immersed in 99% methanol, followed by 100% acetone, and then blocked with 2% goat serum, 2% BSA, and 0.1% saponin in PBS for 1 hour. Cells were incubated with monoclonal anti-FLAG M2 antibody (Sigma-Aldrich) diluted 1:100 in blocking solution for 1 hour, followed by 1-hour incubation with Anti-mouse Alexa Fluor 488 (AF488; Invitrogen) diluted to 1:200 containing DAPI (10 µg/mL). Slides were washed three times with PBS containing 0.01% Tween-20 between incubation steps. Coverslips were mounted with a drop of *SlowFade^TM^* Gold antifade (Life technologies).

Slides were imaged using a Leica DM5500B microscope, equipped with a Hamamatsu 3CCD digital camera operated through Volocity software ver. 6.3 (Quorum Technologies). A fixed exposure of 979 ms was used to detect Alexa Fluor 488 bound to fungal cells, 49 ms used to detect DAPI, and 59 ms for phase contrast. A total of 196 Wos2 and 64 WT cells were measured with 36 fields of view for Wos2 and five fields of view for WT. Following background normalization for fluorescence, 33.67% of the Wos2 cells displayed fluorescence higher than that of WT.

### Murine survival assay and tissue burden analysis

Murine infection assays were performed under the approval of the Animal Utilization Protocol 4193 at the University of Guelph and in accordance with all animal handling guidelines. *C. neoformans* strains (i.e., WT, *wos2*Δ, and *wos2*Δ::WOS2) were grown overnight in YPD at 30°C, sub-cultured overnight at 1:100 in YPD, collected and washed in PBS twice, and resuspended at 4.0 × 10^6^ cells/mL in PBS. Ten female BALB/c elite mice aged 6 to 8-weeks (Charles River Laboratories, ON, Canada) were intranasally inoculated with 50 µL of the *C. neoformans* cell suspension (inoculum of 2 × 10^5^ cells) under isoflurane anesthesia. The mice were monitored daily for signs of morbidity and euthanized by isoflurane and CO_2_ inhalation upon reaching endpoint specifications. Endpoint-determining criteria include loss of 20% total body weight, respiratory issues, or visible signs of neurological deficits. Tissue collection of lungs, bronchoalveolar lavage, and brain was done upon study termination. The collected tissues were weighed and homogenized in 1 mL PBS using a Bullet Blender Storm (Next Advance, Troy, NY, USA). Serial dilutions of the homogenized tissues were plated on the YPD medium supplemented with 32 µg/mL chloramphenicol (provider) and incubated for 48 hours at 30°C. All animal experiments were performed in accordance with the Canadian Council on Animal Care guidelines and approved the University of Guelph’s Animal Care Committee (Animal Utilization Protocol 4193).

## Data Availability

The .RAW and affiliated files are deposited into the publicly available PRIDE partner database for the ProteomeXchange consortium. PRIDE ID: PXD050783.

## SUPPLEMENTAL MATERIAL

Figure S1 spectrum.00152-24-s0001.docx Wos2 supports susceptibility to fluconazole.

Figure S2 spectrum.00152-24-s0002.docx Wos2 supports susceptibility to amphotericin B.

Figure S3 spectrum.00152-24-s0003.docx In vitro characterization of wos2Δ::WOS2.

Figure S4 spectrum.00152-24-s0004.docx In vivo infection characterization of wos2Δ::WOS2.

Table S1 spectrum.00152-24-s0005.xlsx Significant different proteins identified in cellular proteome.

Table S2 spectrum.00152-24-s0006.xlsx Significant different proteins identified in secretome.

Table S3 spectrum.00152-24-s0007.xlsx Strains, plasmids, and primers used in this study.

ASM does not own the copyrights to Supplemental Material that may be linked to, or accessed through, an article. The authors have granted ASM a non-exclusive, world-wide license to publish the Supplemental Material files. Please contact the corresponding author directly for reuse.
