# Supplementary material for: Proteome signatures reveal homeostatic and adaptive oxidative responses by a putative co-chaperone, Wos2, to influence fungal virulence determinants in cryptococcosis
Source: Microbiol Spectr. 2024 Jul 2;12(8):e00152-24. doi: 10.1128/spectrum.00152-24 (PMC11302251; doi:10.1128/spectrum.00152-24)
Supplement: Figure S1 — Wos2 supports susceptibility to fluconazole. [file spectrum.00152-24-s0001.docx]

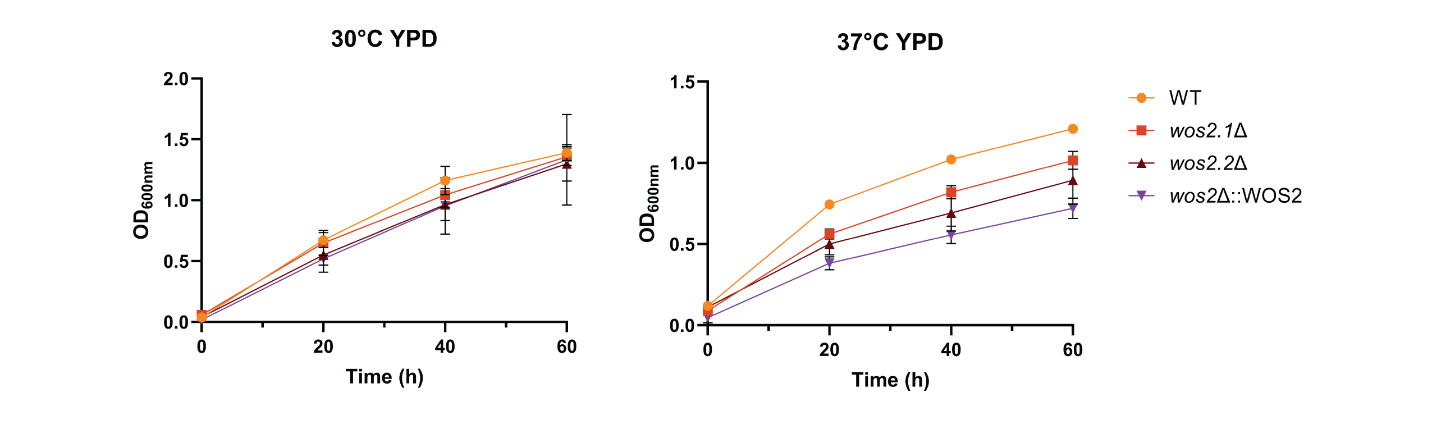


**S1 Fig. Wos2 is important for thermotolerance.** Growth profile of the *wos2*∆ knockout strain had unperturbed growth at 30C and reduced growth at 37C in enriched YPD media compared to WT. Strains were grown overnight in YPD and diluted to an OD_600nm_ of 0.1 in YPD and incubated at 30 or 37 C with OD_600nm_ measurements recorded. Experiment completed in biological triplicate and technical duplicate.
