# Supplementary material for: Proteome signatures reveal homeostatic and adaptive oxidative responses by a putative co-chaperone, Wos2, to influence fungal virulence determinants in cryptococcosis
Source: Microbiol Spectr. 2024 Jul 2;12(8):e00152-24. doi: 10.1128/spectrum.00152-24 (PMC11302251; doi:10.1128/spectrum.00152-24)
Supplement: Figure S2 — Wos2 supports susceptibility to amphotericin B. [file spectrum.00152-24-s0002.docx]

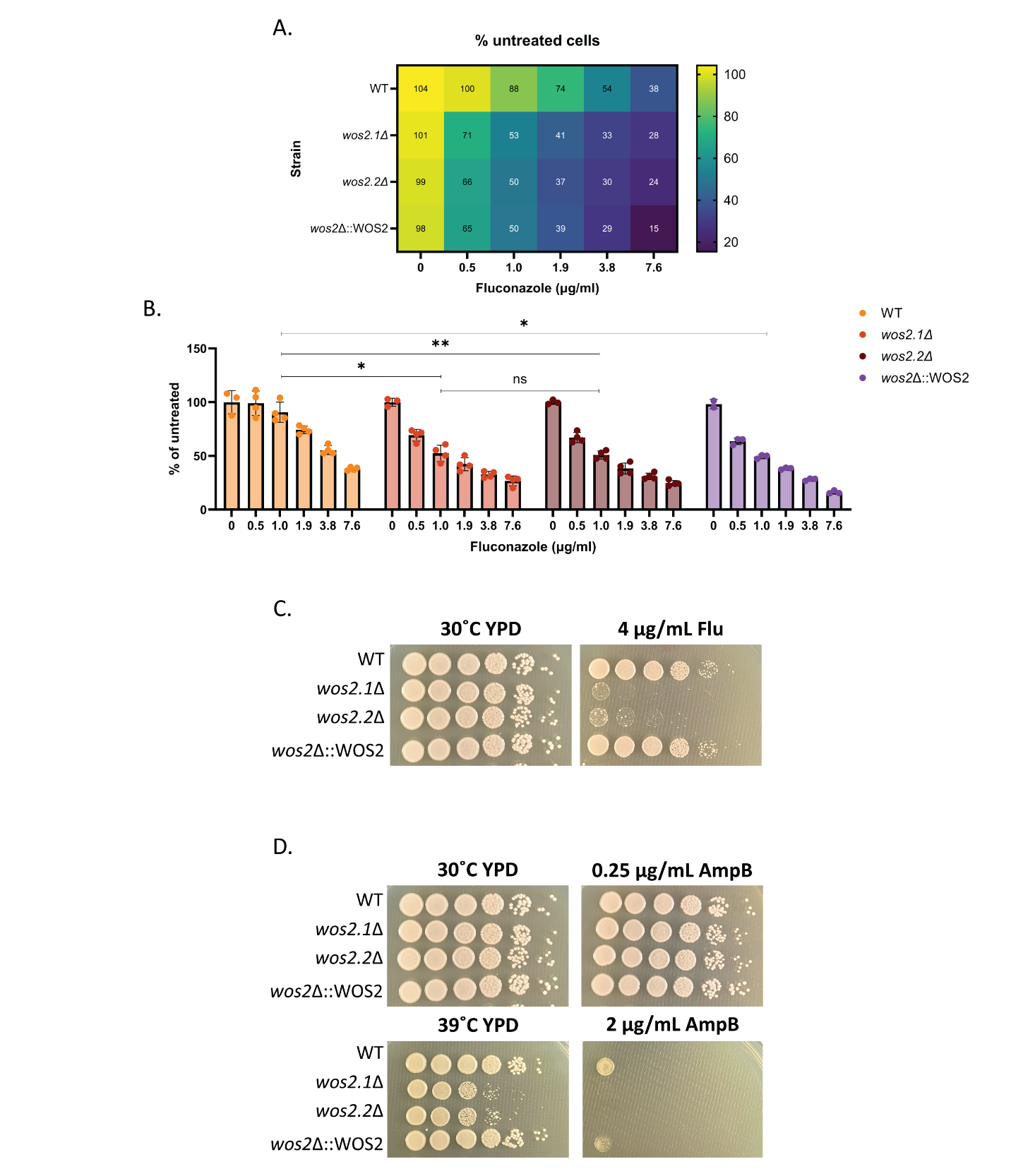


**S2 Fig. Wos2 supports susceptibility to fluconazole and amphotericin B.** A-B) Limiting dilution assay of fluconazole sensitivity to strains at concentrations of 0-7.6 µg/mL. Cell density was measured at OD_600nm ­_and represented as percentage of growth of the untreated control. Data shown representative of one experiment. Experiment completed in biological quadruplicate and technical duplicate. Statistical significance assessed with Tukey’s multiple comparisons test: ns = not significant; *P* < 0.05, *; *P* ≤ 0.005, **. C) Stress agar assay of strains under rich (i.e., YPD) conditions and against 4 µg/mL fluconazole at 30 C for 2 days. D) Stress agar assay of strains under rich conditions and against amphotericin B stress at 30 and 39 C for 2 and 3 days, respectively. Experiment completed in biological duplicate and technical duplicate.
