# Supplementary material for: Proteome signatures reveal homeostatic and adaptive oxidative responses by a putative co-chaperone, Wos2, to influence fungal virulence determinants in cryptococcosis
Source: Microbiol Spectr. 2024 Jul 2;12(8):e00152-24. doi: 10.1128/spectrum.00152-24 (PMC11302251; doi:10.1128/spectrum.00152-24)
Supplement: Figure S3 — In vitro characterization of wos2Δ::WOS2. [file spectrum.00152-24-s0003.docx]

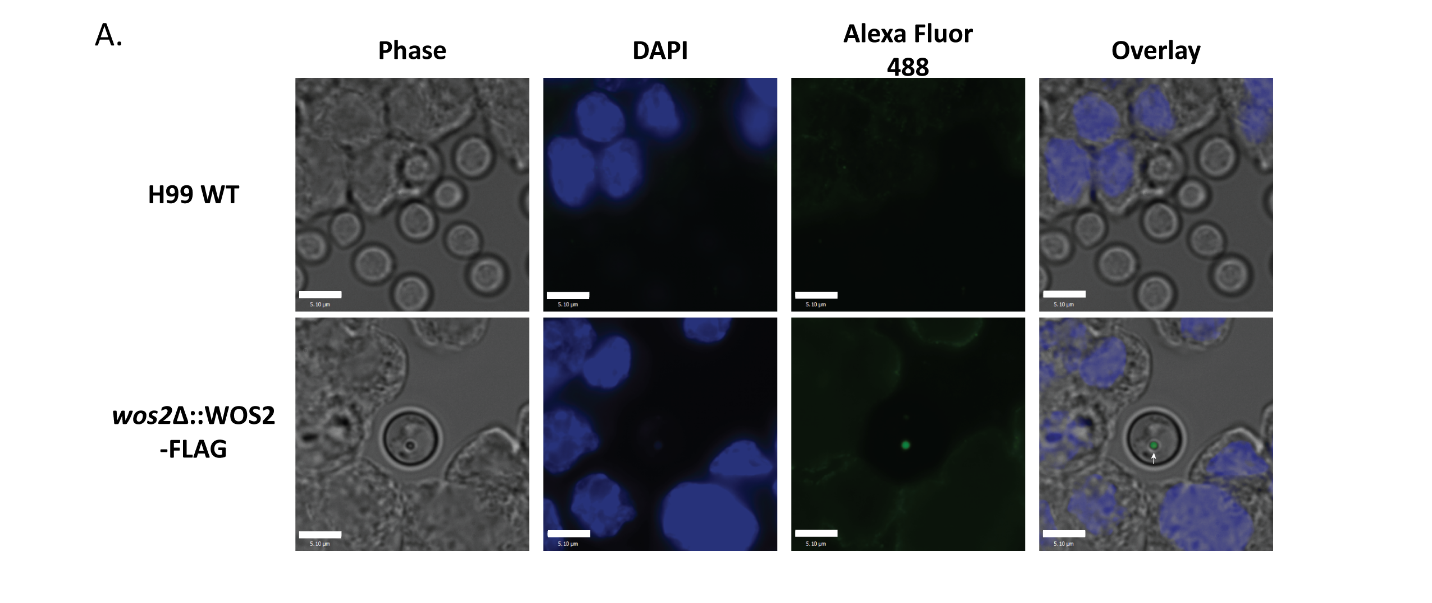


**S3 Fig. *In vitro* characterization of *wos2*Δ::WOS2.** Fluorescence microscopy of *C. neoformans* *wos2*Δ::WOS2-FLAG co-cultured with macrophage (MOI 100:1). Images captured at 3 h.p.i with DAPI and Alexa Fluor 488 (FLAG). Scale bar 5.1 µM. A total of 196 Wos2 and 64 WT cells were measured with 36 fields of view for Wos2 and five fields of view for WT. Following background normalization for fluorescence, 33.67% of the Wos2 cells displayed fluorescence higher than WT.
