# Supplementary material for: Proteome signatures reveal homeostatic and adaptive oxidative responses by a putative co-chaperone, Wos2, to influence fungal virulence determinants in cryptococcosis
Source: Microbiol Spectr. 2024 Jul 2;12(8):e00152-24. doi: 10.1128/spectrum.00152-24 (PMC11302251; doi:10.1128/spectrum.00152-24)
Supplement: Figure S4 — In vivo infection characterization of wos2Δ::WOS2. [file spectrum.00152-24-s0004.docx]

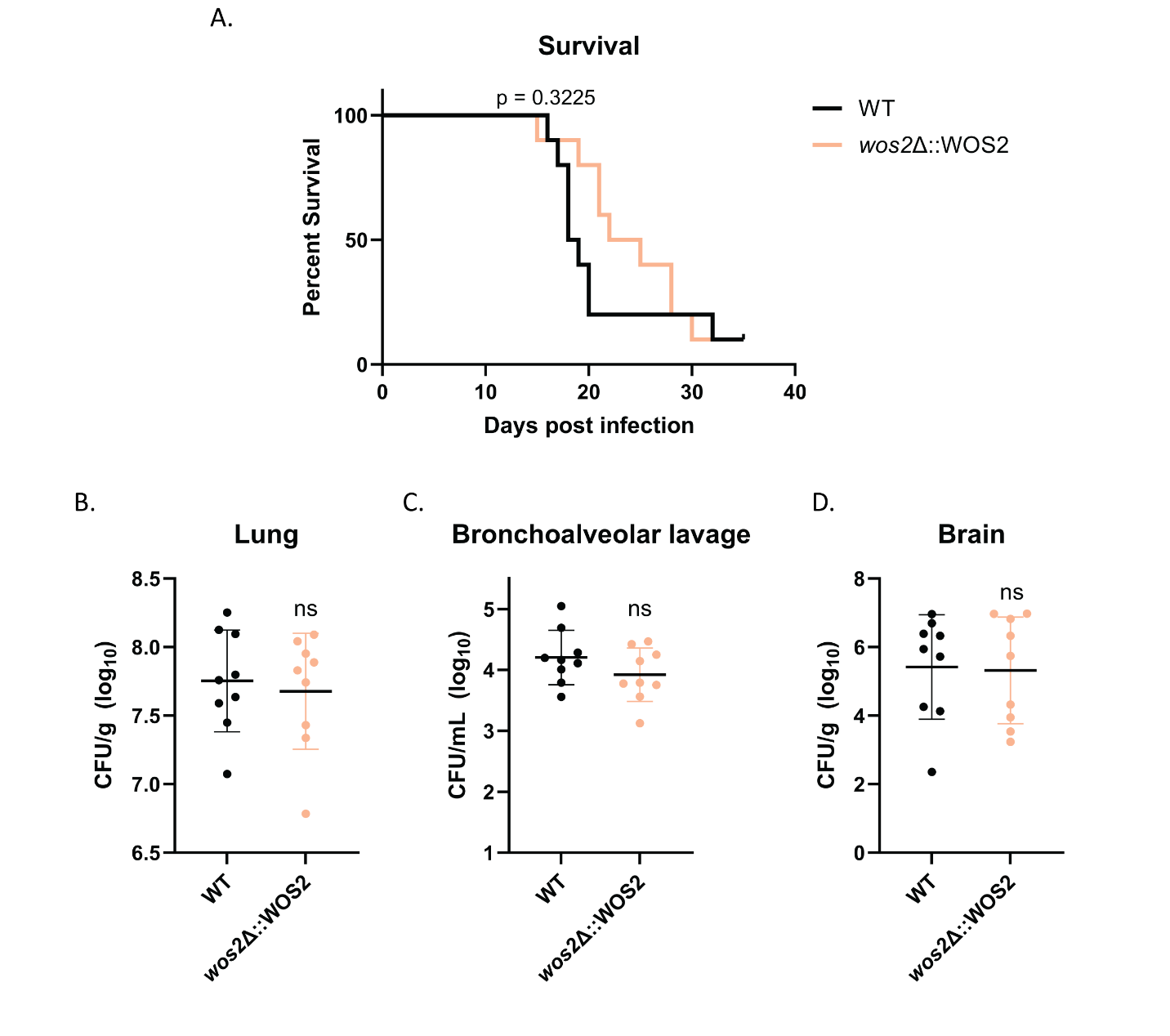


**S4 Fig. *In vivo* infection characterization of *wos2*Δ::WOS2.** A) BALB/c mice infected with *C. neoformans* WT and *wos2*Δ::WOS2 succumbed to infection or survived to assay endpoint (i.e. 35 days). Differences in survival statistically tested using a log-rank (Mantel-Cox) test. Fungal burden from lung (B), bronchoalveolar lavage fluid (C), and brain (D) determined measuring CFUs. Statistical analysis using Students *t*-test.
